# Low-dose *M.tb* infection but not BCG or MTBVAC vaccination enhances heterologous antibody titres in non-human primates

**DOI:** 10.3389/fimmu.2024.1387454

**Published:** 2024-05-10

**Authors:** Marco Polo Peralta Alvarez, Holly Jones, Hugo Redondo Azema, Chloe Davis, Andrew D. White, Charlotte Sarfas, Mike Dennis, Shuailin Li, Daniel Wright, Eugenia Puentes, Simon Kimuda, Sandra Belij-Rammerstorfer, Nacho Aguilo, Carlos Martin, Sally Sharpe, Helen McShane, Rachel Tanner

**Affiliations:** ^1^ Jenner Institute, Nuffield Department of Medicine, University of Oxford, Oxford, United Kingdom; ^2^ Medical Sciences Division, University of Oxford, Oxford, United Kingdom; ^3^ United Kingdom (UK) Health Security Agency, Salisbury, United Kingdom; ^4^ Clinical Research Department y Research and Development Department, Biofabri, Grupo Zendal, Pontevedra, Spain; ^5^ Department of Infectious Diseases, School of Immunology & Microbial Sciences, London, United Kingdom; ^6^ University of Zaragoza, Spanish Network for Research on Respiratory Diseases (CIBERES), Instituto de Salud Carlos III, Madrid, Spain; ^7^ Department of Biology, University of Oxford, Oxford, United Kingdom

**Keywords:** tuberculosis, Mycobacterium tuberculosis, BCG, MTBVAC, antibodies, heterologous immune responses

## Abstract

**Introduction:**

Mycobacteria are known to exert a range of heterologous effects on the immune system. The mycobacteria-based Freund’s Complete Adjuvant is a potent non-specific stimulator of the immune response used in immunization protocols promoting antibody production, and Mycobacterium bovis Bacille Calmette Guérin (BCG) vaccination has been linked with decreased morbidity and mortality beyond the specific protection it provides against tuberculosis (TB) in some populations and age groups. The role of heterologous antibodies in this phenomenon, if any, remains unclear and under-studied.

**Methods:**

We set out to evaluate antibody responses to a range of unrelated pathogens following infection with Mycobacterium tuberculosis (M.tb) and vaccination with BCG or a candidate TB vaccine, MTBVAC, in non-human primates.

**Results:**

We demonstrate a significant increase in the titer of antibodies against SARS-CoV-2, cytomegalovirus, Epstein-Barr virus, tetanus toxoid, and respiratory syncytial virus antigens following low-dose aerosol infection with M.tb. The magnitude of some of these responses correlated with TB disease severity. However, vaccination with BCG administered by the intradermal, intravenous or aerosol routes, or intradermal delivery of MTBVAC, did not increase antibody responses against unrelated pathogens.

**Discussion:**

Our findings suggest that it is unlikely that heterologous antibodies contribute to the non-specific effects of these vaccines. The apparent dysregulation of B cell responses associated with TB disease warrants further investigation, with potential implications for risk of B cell cancers and novel therapeutic strategies.

## 1 Introduction

Tuberculosis (TB), caused by *Mycobacterium tuberculosis* (*M.tb*), remains a disease of global public health importance, with a quarter of the world’s population estimated to be infected and over 10 million new cases every year (1). Mycobacteria have long been known to have heterologous or non-specific effects on the immune system. For example, Complete Freund’s Adjuvant, composed of heat-killed and dried *M.tb* (strain H37Ra), is the most widely-used and effective adjuvant for experimental antibody production (2, 3). Furthermore, *Mycobacterium bovis* Bacille Calmette Guérin (BCG) vaccination has been associated with reduced incidence of non-tuberculous infectious disease in adolescents and a reduction in all-cause mortality in infants (4–8). It continues to be used to treat non-muscle-invasive bladder cancer, and has been for more than 30 years (9). The mechanisms underlying these effects are not well-understood, and non-specific immune activation may be a double-edged sword with both beneficial and detrimental effects dependent on the context (10, 11).

The non-specific effects of BCG vaccination have been largely attributed to epigenetic and metabolic reprogramming of innate (or innate-like) cells such as monocytes, natural killer cells, neutrophils and γδ T cells that allows them to respond more effectively to antigen exposures unrelated to the original stimulus (12–15). There is a growing body of literature supporting this ‘trained immunity’ mechanism, although previous studies also indicate a potential role for cross-reactive T cells or BCG-induced proinflammatory cytokine release by lymphocytes acting non-specifically to activate bystander macrophages, resulting in a state of temporarily heightened innate immunity (16–19). Regarding humoral immunity, IgG titres against hepatitis B, polio, pneumococcal capsular polysaccharide antigens, *H.influenzae* type b polysaccharide and tetanus toxoid, induced after their respective vaccines, tend to be higher in BCG-vaccinated individuals (20–22). The potential for BCG to induce autoantibodies is equivocal, although IgG recognising host peptides has been reported following BCG vaccination in healthy adults (23, 24).

*M.tb* infection itself may similarly enhance antibodies to unrelated pathogens, although their functional relevance is unclear given the concomitant activation of immunoregulatory circuits and associated epidemiological evidence for increased susceptibility to other infections in TB patients (25, 26). Conventional B cell activation occurs via cognate interaction with T follicular helper cells, thus ensuring specificity of the antibody response against the activating target and acting as a safeguard against undesired and potentially detrimental B cell responses (27). However, there are alternative mechanisms that can lead to non-specific, or polyclonal, activation of B cells and result in antibody responses unrelated to the initial stimulus (28). Such a phenomenon may play an advantageous role in the maintenance of serological memory (29), but could also turn on anti-self responses and lead to autoimmune manifestations during chronic infections (28, 30). Furthermore, polyclonal activation of memory B cells in malaria infection has been linked to an increased risk of Burkitt’s lymphoma (31). Given that B cell dysregulation has been reported in TB patients (32), as well as an increased risk of B cell lymphomas (33–36), an improved understanding of this phenomenon is critical.

MTBVAC is one of the leading TB vaccine candidates and is currently in Phase 3 clinical trials (37). It is based on the rational attenuation of *M.tb* with two stable and independent genetic deletions located in the *phoP* and *fadD26* genes. It thus has a PhoP-/PDIM-deficient phenotype while maintaining the rest of the antigens present in *M.tb* but absent in BCG (38). Similar to BCG, vaccination with MTBVAC has been shown to induce trained innate immunity with epigenetic reprogramming of human monocytes, and it confers protection against lethal pneumonia in mice (39). Given that it represents an attenuated form of *M.tb*, it would be of interest to determine whether MTBVAC vaccination results in polyclonal memory B cell activation and production of antibodies against heterologous pathogens, and whether this contributes to the non-specific effects observed.

The current study aimed to determine whether infection with *M.tb*, or vaccination with BCG or MTBVAC, influences antibody responses to a range of unrelated pathogens. Kimuda et al. have previously shown that active pulmonary TB patients in Uganda had higher antibody responses to respiratory syncytial virus (RSV) and measles virus (MV) compared with uninfected controls, while BCG vaccination in uninfected UK adults did not alter levels of antibodies against heterologous pathogens (40). There were some limitations to this study including inability to measure matched pre- and post-*M.tb* infection responses in the same individuals, and the unknown time after infection that samples were collected. Furthermore, in the BCG vaccinated cohort, responses were measured at a relatively early timepoint of three weeks post-vaccination. We aimed to validate these findings and address the shortcomings by using a more controlled experimental *M.tb* infection model in non-human primates (NHPs). Here the infectious *M.tb* dose and sampling timepoints are standardized, exposure to other pathogens and other confounders are minimal, and the responses measured can be matched pre- and post-infection. Furthermore, we evaluated responses to BCG vaccination in naïve animals for a longer period post-vaccination (at weeks 8 and 20). We then expanded on the previous study by also measuring IgM, evaluating different routes of BCG administration (intradermal, intravenous and aerosol), and by exploring heterologous antibody responses following MTBVAC vaccination.

## 2 Methods

### 2.1 Non-human primate studies

#### 2.1.1 Experimental animals

Rhesus macaques of Indian genotype aged 3-6 years (Studies 1, 2 and 4) or 10-12 years (Study 3) were obtained from an established UK breeding colony. Absence of previous exposure to mycobacterial antigens was confirmed by *ex-vivo* IFN-γ ELISpot (MabTech, Nacka. Sweden) to measure responses to mycobacterial antigens: purified protein derivative (PPD) (SSI, Copenhagen, Denmark), and pooled 15-mer peptides of ESAT-6 and CFP-10 (Peptide Protein Research LTD, Fareham, U.K.) on three occasions over a minimum of six weeks prior to vaccination or *M.tb* challenge. Animals were housed in compatible social groups, in accordance with the Home Office (UK) Code of Practice for the Housing and Care of Animals Used in Scientific Procedures (1989), and the National Committee for Refinement, Reduction and Replacement (NC3Rs), Guidelines on Primate Accommodation, Care and Use, August 2006 (NC3Rs, 2006).

Animals were sedated by intramuscular injection of ketamine hydrochloride (Ketaset, 100mg/ml, Fort Dodge Animal Health Ltd, Southampton, UK; 10mg/kg) for procedures requiring removal from their housing. None of the animals had been used previously for experimental procedures and each socially compatible group was randomly assigned to a particular study treatment. All animal procedures and study design were approved by the UK Health Security Agency (and predecessor organisation Public Health England), Porton Down Ethical Review Committee, and authorized under an appropriate UK Home Office project license. Stored serum samples were used from five historical NHP studies to avoid the use of additional animals.

#### 2.1.2 *M.tb* challenge

In Study 1, serum was collected from BCG-naïve animals at baseline and 12-16 weeks post experimental infection with <10-20 colony forming units (CFU) *M.tb* Erdman strain K01 (BEI Resources) via the natural aerosol route (n=16). Mono-dispersed bacteria in particles were generated using a 3-jet collison nebuliser (BGI) and, in conjunction with a modified Henderson apparatus, delivered to the nares of each sedated primate via a modified veterinary anaesthesia mask. Challenge was performed on sedated animals placed within a ‘head-out’, plethysmography chamber (Buxco, Wilmington, North Carolina, USA). Measures of disease severity used in this analysis were lesion count and computed tomography (CT) score at 3 weeks (available for n=16 animals), 6 weeks (n=5), 8 weeks (n=16), and 12 weeks (n=11) post-challenge.

#### 2.1.3 BCG vaccination

BCG vaccinations were performed using BCG Danish strain 1331 (AJ Vaccines, Denmark), prepared according to the manufacturer’s instructions by addition of 1 ml Sauton’s diluent (for intradermal and intravenous delivery) or 1 ml of sterile saline (for aerosol delivery) to a lyophilised vial. Animals received either 2–8 × 10^5^ CFU of BCG intradermally (ID) into the upper arm under sedation (Study 2, n = 7); 2–8 × 10^6^ CFU of BCG intravenously (IV) into the femoral vein of the left leg (Study 3, n = 6); or 1 x 10^7^ CFU of BCG delivered to the lungs using an Omron MicroAir mesh nebuliser (Omron Healthcare United Kingdom Ltd., Milton Keynes, United Kingdom) with a mouthpiece attachment (nares occluded) or a modified paediatric anaesthesia mask attachment (Study 4, n=8). Serum was collected at baseline and at 8 and 20 weeks post-ID BCG vaccination, 8 weeks post-IV BCG vaccination, and 4 and 12 weeks post-aerosol BCG vaccination.

#### 2.1.4 MTBVAC vaccination

In Study 5, serum was collected from animals that received ID vaccination with 3–17 × 10^5^ CFU of MTBVAC into the upper arm under sedation (Biofabri, Spain) (n=8) as previously described (41). Serum was collected at baseline and at 8 and 20 weeks post-MTBVAC vaccination.

### 2.2 Indirect enzyme-linked immunosorbent assays

Antigen-specific IgM and IgG levels were determined by indirect ELISA. The antigens used were *M.tb* PPD (AJ Vaccines), *Salmonella abortus* (*S. abortus*) lipopolysaccharide (LPS) (Enzo Life Sciences), recombinant SARS-CoV-2 spike protein S1 subunit (RayBiotech), tetanus toxoid (TT) (Cayman Chemical), cytomegalovirus (CMV) native antigen (BIOZOL), Epstein-Barr virus (EBV) extract antigen (BIOZOL), measles morbillivirus (MV) antigen (BIOZOL), *E. coli* (strain O157) antigen (BIOZOL) and respiratory syncytial virus (RSV) antigen (RayBiotech). NUNC MaxiSorp flat-bottom immuno-microwell plates (44-2404, Thermo Fisher Scientific) were coated with 50µl of antigen at a concentration of 5µg/ml and incubated for 16-18 hours at 4°C. After this period, plates were washed four times with Dulbecco’s phosphate-buffered saline with 0.05% tween (DPBS-T). After 1 hour incubation with 100µl per well of casein blocker (37528, Thermo Fisher Scientific) at 20°C, blocker was flicked off and 50µl of serum diluted 1:100 in casein was added to wells in triplicate. Two positive controls (known high antibody responders) and a negative control (casein-only) were also included. After a 2-hour incubation at room temperature, plates were washed six times with DPBS-T. 50µl of alkaline phosphatase conjugated secondary antibody (µ chain-specific goat anti-monkey IgM or γ chain-specific goat anti-monkey IgG, Rockland Laboratories) diluted to 5µg/ml in casein was added to each well and incubated for one hour. During incubation, the p-Nitrophenyl Phosphate (pNPP) substrate was prepared using pNPP tablets (34047, Thermo Fisher Scientific) dissolved in H_2_O and 5x diethanolamine (DEA) buffer (34064, Thermo Fisher Scientific). After incubation, plates were washed 6 times with DPBS-T and 50µl of developing solution was added to each well. Sample absorbance was read at OD_405_ using a BioTek-2 ELISA plate reader.

### 2.3 Avidity ELISAs

96-well NUNC high protein binding MaxiSorp plates (44-2404, Thermo Fisher Scientific) were coated with 50µl of antigen solution diluted in DPBS, and incubated for 16-18 hours at 4°C. Plates were then blocked with 100µl of Casein for 1 hour. Block solution was flicked off and 50µl of serum diluted 1:100 in casein was added to wells in triplicate. The antigens used were: *M.tb* whole cell lysate (WCL) 1µg/ml, *M.tb* PPD 5µg/ml, SARS-CoV-2 S1 5µg/ml, CMV native antigen 5µg/ml, EBV extract antigen 5µg/ml and RSV antigen 5µg/ml. Plates were then incubated for 2 hours with 50µl of a variable concentration (calculated to achieve an initial optical density of ~1) of NHP serum diluted in casein. 50µl of the chaotropic agent sodium thiocyanate (NaSCN) (Sigma Aldrich) was added in increasing concentrations to the samples and incubated for 15 minutes at room temperature. In all cases NaSCN concentrations ranged from 0M to 4M. Plates were then incubated for 1 hour with 50µl per well of 1:1000 dilution in casein of goat anti-monkey IgG γ-chain-specific secondary antibody conjugated to alkaline phosphatase (AP) (Rockland Laboratories). Finally, 100µl of pNPP substrate developing solution (made as described in 2.2) was added. In the case of the *M.tb* WCL experiments, plates reached an initial optical density of 1 after approximately 1 hour. In the case of heterologous antigens, plates were left overnight at 4°C before reading at a wavelength of 405 nm with a BioTek-2 plate reader. Plates were washed 4x in PBS-Tween and tapped dry between each step, except after blocking where washing was not performed. For each sample, the response was normalised from 0 to 100% using GraphPad Prism software, followed by calculation of the IC_50_ value.

### 2.4 Statistical analysis

A Shapiro Wilk normality test was performed for all data sets. For samples comparing three time points, parametric one-way ANOVAs were used for analysis of data with a normal distribution, and a non-parametric Friedman’s test was used for those not normally distributed. For data with two time points, a paired t-test was used for normally distributed data, and a Wilcoxon test was used for non-normal data. *Post-hoc* tests were carried out to correct for multiple comparisons; a Dunn’s test was used after the Freidman’s tests, and a Dunnett’s test after ANOVAs. To investigate correlations, two-tailed Spearman’s rank correlation tests were performed using IBM SPSS Statistics Version 29.0. Figures were plotted using R version 4.0.1.

## 3 Results

### 3.1 Low-dose *M.tb* infection enhances heterologous antibody responses which are associated with TB disease severity

**Figure 1:**
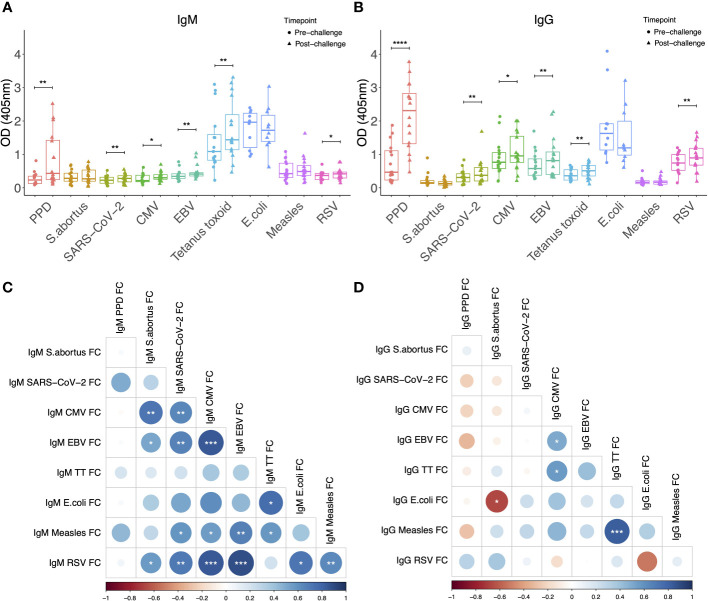
IgM **(A)** and IgG **(B)** levels to different pathogen antigens in the sera of rhesus macaques collected at baseline and 12-16 weeks post-low dose M.tb challenge. * denotes p<0.05, ** denotes p<0.01, *** denotes p<0.001, **** denotes p<0.0001. Error bars represent the median and interquartile range (IQR). Spearman’s correlations between heterologous IgM **(C)** and IgG **(D)** responses to different pathogen antigens. OD, optical density; PPD, purified protein derivative; CMV, cytomegalovirus; EBV, Epstein Barr virus; RSV, respiratory syncytial virus; FC = fold change in antibody response between pre- and post-M.tb challenge.

Sera from NHPs was evaluated prior to and at 12-16 weeks post-challenge with <10-20 CFU of *M.tb* by the aerosol route. As expected, levels of both IgM and IgG specific to PPD increased significantly post-*M.tb* infection (p = 0.0003 and p < 0.0001 respectively), confirming induction of a specific antibody response (**Figures 1A, B**). There were also modest but significant increases post-infection in both IgM and IgG specific for SARS-CoV-2 spike protein (p = 0.048 and p = 0.023 respectively), CMV (p = 0.006 and p = 0.007 respectively), EBV (p = 0.002 and p = 0.003 respectively), tetanus toxoid (p = 0.001 and p = 0.002 respectively), and RSV (p = 0.022 and p = 0.0035 respectively) (**Figures 1A, B**). There were several significant correlations between IgM responses to different pathogen antigens (**Figure 1C**), but fewer correlations between IgG responses to different pathogen antigens (**Figure 1D**). Levels of heterologous responses to unrelated pathogen antigens did not correlate with the specific response to PPD for either isotype.

**Figure 2:**
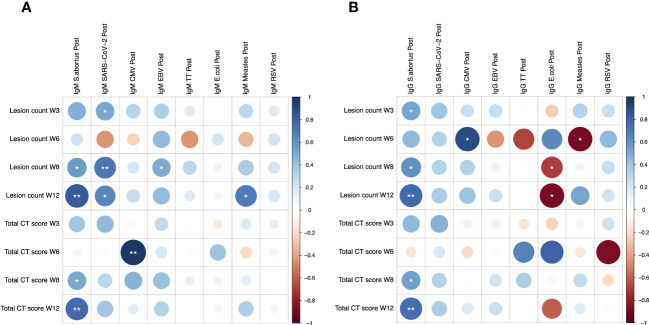
Spearman’s correlations between heterologous IgM **(A)** and IgG **(B)** responses to different pathogen antigens and pathology following low dose M.tb challenge as measured by lung lesion count and CT score. * denotes p<0.05, ** denotes p<0.01. CT, computerized tomography; CMV, cytomegalovirus; EBV, Epstein Barr virus; TT, tetanus toxoid; RSV, respiratory syncytial virus.

Potential associations between heterologous antibody responses and pathology following *M.tb* infection were explored. *S. abortus*-specific IgM was significantly correlated with lung lesion counts at weeks 8 and 12 (r = 0.569 and 0.832, p = 0.022 and 0.001 respectively), and with CT score at weeks 8 and 12 (r = 0.515 and 0.767, p = 0.041 and 0.006 respectively) (**Figure 2A**). Similarly, *S. abortus*-specific IgG was significantly correlated with lung lesion counts at weeks 3, 8 and 12 (r = 0.523, 0.602 and 0.779, p = 0.038, 0.014 and 0.005 respectively), and with CT score at weeks 8 and 12 (r = 0.534 and 0.746, p = 0.033 and 0.008 respectively) (**Figure 2B**). CMV-specific IgM was associated with CT score at week 6 (r = 0.975, p = 0.005) (**Figure 2A**), and CMV-specific IgG was correlated with lung lesion count at week 6 (r = 0.894, p = 0.041) (**Figure 2B**). SARS-CoV-2-specific IgM was also correlated with lung lesion counts at weeks 3, 8 and 12 (r = 0.519, 0.710 and 0.644, p = 0.040, 0.002 and 0.033 respectively); EBV-specific IgM was associated with lung lesion count at week 8 (r = 0.505, p = 0.046); and MV-specific IgM was associated with lung lesion count at week 12 (r = 0.669, p = 0.017) (**Figure 2A**).

### 3.2 Low-dose *M.tb* infection does not enhance avidity of IgG against unrelated pathogens

**Figure 3:**
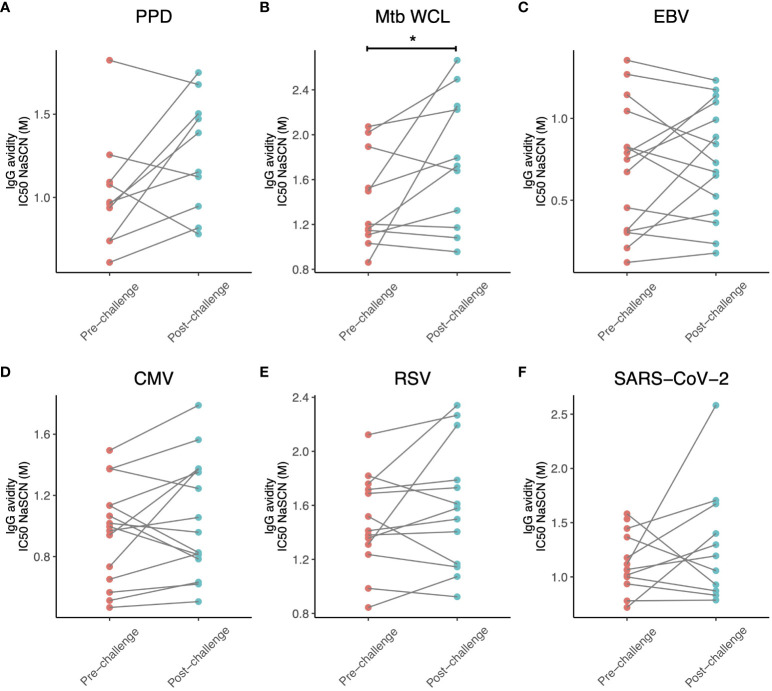
Avidity of IgG specific for **(A)** PPD, **(B)** *M.tb* WCL, **(C)** EBV, **(D)** CMV, **(E)** RSV and **(F)** SARS-CoV-2 in the sera of rhesus macaques collected at baseline and 12-16 weeks post-low dose M.tb challenge. * denotes p<0.05. PPD, purified protein derivative; CMV, cytomegalovirus; EBV, Epstein Barr virus; RSV, respiratory syncytial virus.

We then assessed whether IgG avidity was influenced by *M.tb* infection for some of the antigens against which antibody titres were increased (SARS-CoV-2, CMV, EBV and RSV). There was a modest but statistically significant increase in *M.tb* WCL-specific IgG avidity (p = 0.049, **Figure 3B**), but no differences for any of the other antigens (**Figures 3A, C–F**), although there was a trend towards increased avidity of CMV-specific and RSV-specific IgG (p = 0.22 and 0.20 respectively). In both cases, 9 out of 13 animals tested showed an increase in IgG avidity following M.tb infection (**Figures 3E, F**). There was a significant positive correlation between *M.tb* WCL-specific IgG avidity following *M.tb* infection, or fold change in IgG avidity, and CT score at week 8 (r = 0.627 and 0.755, p = 0.039 and 0.007 respectively). Changes in avidity of IgG specific to the other antigens tested did not associate with pathology.

### 3.3 BCG vaccination administered by the ID, IV or aerosol route does not enhance antibody responses against unrelated pathogens

**Figure 4:**
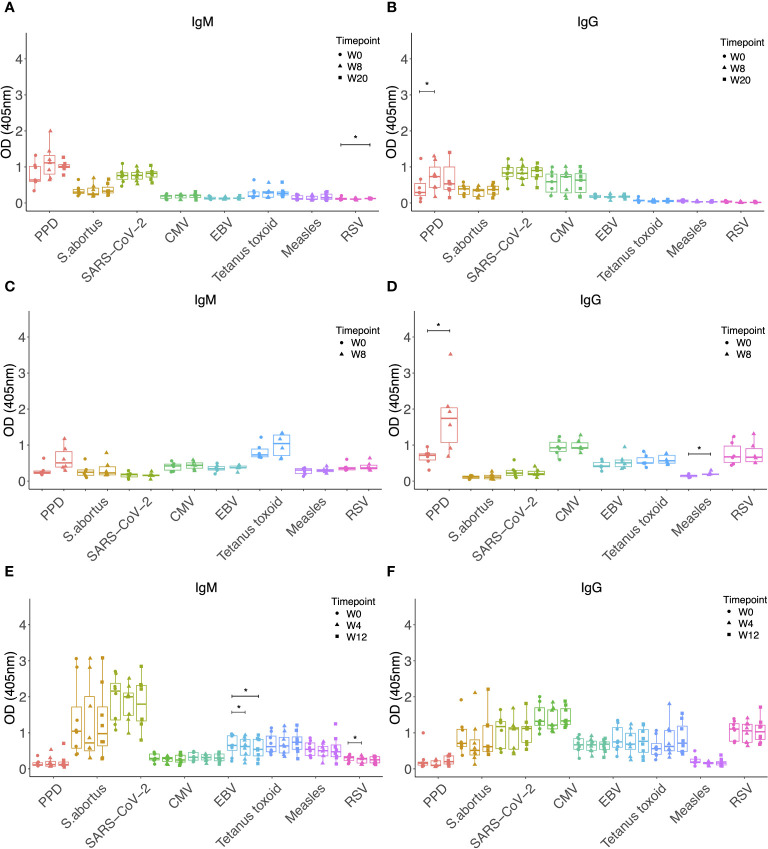
IgM **(A)** and IgG **(B)** levels to different pathogen antigens in the sera of rhesus macaques collected at baseline and 4, 8 and 20 weeks post ID BCG vaccination. IgM **(C)** and IgG **(D)** levels to different pathogen antigens in the sera of rhesus macaques collected at baseline and 8 weeks post IV BCG vaccination. IgM **(E)** and IgG **(F)** levels to different pathogen antigens in the sera of rhesus macaques collected at baseline and 4 and 12 weeks post aerosol BCG vaccination. * denotes p<0.05. Error bars represent the median and IQR. OD, optical density; PPD, purified protein derivative; CMV, cytomegalovirus; EBV, Epstein Barr virus; RSV, respiratory syncytial virus.

To determine whether a similar effect of increased antibody responses against heterologous pathogens could be observed following BCG vaccination, we evaluated samples taken prior to and at 8 and 20 weeks post-BCG vaccination by the standard ID route. Neither IgM nor IgG responses to any of the antigens tested increased, other than a significantly increased titre of PPD-specific IgG as expected (p = 0.015, **Figures 4A, B**). Given the higher specific immunogenicity associated with BCG vaccination by the IV route, and the ten-fold higher vaccine dose administered in this study compared with ID BCG, we hypothesized that we may be more likely to observe increases in heterologous antibody responses following IV BCG vaccination. However, while there was an increase in PPD-specific IgG (p = 0.026), enhancement of IgM or IgG responses was not seen to any of the heterologous antigens, with the exception of MV-specific IgG at 8 weeks post-IV BCG (p = 0.031, **Figures 4C, D**).

We hypothesized that the disparity in the effect on antibody responses to heterologous pathogens between *M.tb* infection and BCG vaccination may be due to the aerosol route of *M.tb* infection as opposed to systemic delivery of BCG vaccination. We therefore evaluated samples collected at baseline and at 4 and 12 weeks following BCG vaccination by the aerosol route. Neither IgM nor IgG to any of the antigens tested significantly increased after aerosol vaccination, and in fact there was a decrease in IgM responses to TT and RSV (**Figures 4E, F**).

### 3.4 MTBVAC vaccination does not enhance antibody responses against unrelated pathogens

**Figure 5:**
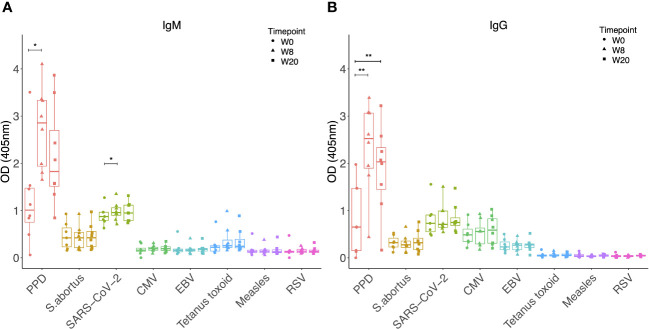
IgM **(A)** and IgG **(B)** levels to different pathogen antigens in the sera of rhesus macaques collected at baseline and 4, 8 and 20 weeks post ID MTBVAC vaccination. * denotes p<0.05, ** denotes p<0.01. Error bars represent the median and IQR. OD, optical density; PPD, purified protein derivative; CMV, cytomegalovirus; EBV, Epstein Barr virus; RSV, respiratory syncytial virus.

Finally, we investigated whether vaccination with MTBVAC, which is an attenuated strain of *M.tb* and therefore more similar to *M.tb* than BCG, could result in increased antibody responses to heterologous pathogens in a similar manner to *M.tb* infection. MTBVAC vaccination did not enhance IgM or IgG responses to any of the non-mycobacterial antigens tested, other than a modest increase in SARS-CoV-2 specific IgM at 8 weeks post-vaccination (p = 0.04, **Figure 5A**). There were significantly increased IgM (p = 0.013 at week 8, **Figure 5A**) and IgG (p = 0.003 at week 8 and p = 0.004 at week 20, **Figure 5B**) responses to PPD as expected, and these were more potent than the specific responses induced by BCG vaccination alone (**Figures 1A, B**).

## 4 Discussion

Our study demonstrates the significant enhancement of antibody titres against heterologous pathogens following low-dose *M.tb* challenge in NHPs. This is consistent with the findings of Kimuda et al., who observed higher heterologous antibody responses in patients with pulmonary TB disease compared with uninfected controls in Uganda, and provides validation of this effect in a more controlled system that enables the direct comparison of matched pre- and post-infection samples (40). Once the human data were adjusted for potential confounders, active TB was associated with higher levels of anti-RSV and anti-measles antibodies compared to the uninfected individuals (40). We also observed an increase in RSV-specific antibodies, although anti-measles responses were not altered in the NHPs. If the hypothesis regarding polyclonal activation of B cells as an underlying mechanism stands, this is consistent with high levels of seropositivity to RSV in captive breeding macaques (42, 43), and the presence of a memory B cell pool specific for measles in humans [due to its inclusion in the Expanded Programme on Immunization (EPI) vaccine schedule] but not NHPs. Other pathogens which NHPs frequently encounter including the NHP homologues of CMV and EBV showed significant increases in antibody levels following *M.tb* challenge in our study (44, 45). However, responses to tetanus toxoid, against which the NHPs were not vaccinated, also increased. Exposure to *Clostridium tetani* in their environment cannot be ruled out (46), although natural exposure is unlikely to induce antibodies to the toxin given that the lethal dose is lower than the threshold required for immunogenicity. Alternatively, antibodies raised against other pathogens the animals have been exposed to may cross-react with tetanus toxoid, as described in responses to certain chlamydial proteins among others (47).

The inclusion of both IgM and IgG allowed us to explore whether the effects of *M.tb* infection on heterologous antibodies are isotype-specific. It is noteworthy that increases were seen in both isotypes against a range of pathogen antigens, although the mechanisms likely differ. Analysis of associations between responses to different pathogens within each isotype revealed many more significant correlations for IgM than IgG. This is perhaps unsurprising given that IgM is derived from germinal centre (GC)-independent subsets of memory B cells and is therefore less specific to its cognate antigen (48). Alternatively, the IgM responses could be caused by upregulation of non-specific natural IgM production by B-1 cells (49). IgG, on the other hand, is derived from memory B cells which have undergone class switching and affinity maturation in the GC of lymph nodes and are expected to have high specificity (48). It is therefore more difficult to account for our observed increases in heterologous IgG responses. The lack of any significant homology between *M.tb* and the pathogens tested renders classical cross-reactivity unlikely. However, IgG+ memory B cells that can be formed in a GC-independent manner with low levels of somatic hypermutation have been previously described (50). Future studies of experimental *M.tb* infection in germ-free animal models could better elucidate underlying mechanisms.

The samples used in this study were from NHPs that received an ultra-low to low challenge dose of *M.tb*. We selected these conditions as they most closely resemble those of natural infection while still inducing measurable disease burden in all exposed animals that could be related to levels of heterologous antibodies (51). It is striking that we are still able to detect a significant increase in heterologous antibody levels despite the very low dose of *M.tb* administered. We also observed a significant correlation between levels of pathology following *M.tb* challenge and magnitude of heterologous antibody responses. This suggests that the effect may be associated with antigen load and/or disease severity. *M.tb* challenge is known to induce interleukin-21 (IL-21), which is significantly upregulated in TB granulomas and essential for control of infection (52). Given that IL-21 can be a potent driver of memory B cell differentiation into antibody-secreting cells upon CD40 ligation (independent of BCR signalling) (53, 54), it is possible that local production of IL-21 together with increased expression of CD40L on activated T cells in the inflammatory environment of the diseased lung promotes differentiation of heterologous plasma cells leading to the production of antibodies against unrelated pathogens.

Levels of other proinflammatory cytokines induced by *M.tb* such as interferons and interleukin-6 (IL-6) also promote the activation of B cells, their differentiation into plasmablasts and antibody secretion (55). An increased mycobacterial load would result not only in more mycobacterial proteins and nucleic acids that can act on B cell Toll-like receptors and other pathogen-recognition receptors independently of the BCR (29), but also resultant increases in these cytokines. Indeed, increased levels of IL-6 have been associated with lung cavitation, while IFN-γ and TNF-α are positively associated with disease severity and bacterial burden in the lung (56, 57). It would be interesting to determine whether a higher challenge dose confers a stronger heterologous response, and whether total IgM and IgG levels, rather than antigen-specific, are enhanced by *M.tb* infection and correlate with disease severity as this may offer increased sensitivity to detect an association. Another key area for future research is to determine whether this phenomenon is unique to *M.tb* or a subset of virulent pathogenic infections, or is merely a by-product of general inflammation and immune activation. Similar effects have been described in the context of malaria, hepatitis C, Chagas disease, HIV infection and hantavirus pulmonary disease (31, 58, 59), although the underlying mechanisms may be different.

That BCG vaccination did not have any effect on heterologous antibodies in this study is again consistent with the findings of Kimuda et al. in UK adults (40). This is despite evaluating samples collected over a longer follow-up period post-vaccination. Given the high degree of homology between *M.tb* and BCG (60), it is perhaps surprising that heterologous antibodies are enhanced by one mycobacterial species and not the other. We thus explored some of the variables differing between the two studies to better understand this disparity. Firstly, *M.tb* challenge is a more potent immune stimulator resulting in an actively replicating mycobacterial infection that reaches a higher antigen load compared with the local, self-limited replication seen following ID BCG vaccination. We therefore evaluated serum from animals vaccinated intravenously with a ten-fold higher dose of BCG, which is known to be more potently immunogenic than the standard ID regimen (61, 62). However, when this also failed to enhance heterologous antibodies, we considered that the aerosol route of administration may be key. Still, aerosol BCG vaccination failed to boost responses despite a markedly higher administered dose compared with low-dose *M.tb* infection. Overall this suggests that BCG vaccination does not induce antibodies against unrelated pathogens, and that antibodies do not contribute to the non-specific effects of BCG vaccination described in the literature, which are likely rather mediated by trained innate immunity (13).

Finally, we reasoned that the effects on heterologous antibodies could be mediated by antigens present in *M.tb* but not BCG. As an intermediate, we explored vaccination with the attenuated *M.tb* vaccine candidate, MTBVAC, but again did not find any observable effects. This may be due to the lack of essential virulence factors that are present in *M.tb* but not MTBVAC, including those encoded by the phoP and fadD26 genes (63), the small sample sizes (n=8 animals), or the use of an intradermal rather than aerosol route of administration; it would be of interest to quantify heterologous antibody responses following aerosol MTBVAC vaccination in future studies (64). Alternatively, it suggests that the increased titres of heterologous antibodies is mediated by mycobacterial infection but not vaccination, which is perhaps reassuring given the potential detrimental impacts of polyclonal activation of memory B cells (28, 30), should that be the applicable mechanism. It was interesting to note the significantly higher *M.tb*-specific IgM and IgG responses following MTBVAC (**Figure 5**) compared with BCG vaccination (**Figure 4**). Further work is required to better characterize the antibody response to MTBVAC and understand whether any qualities contribute to the superior protection conferred, particularly given that mucosal MTBVAC vaccination in rhesus macaques induced IgG and IgM in the bronchoalveolar lavage that had increased binding to *M.tb* and increased antibody-dependent phagocytosis by THP-1 cells *in vitro* (64).

One finding in our study that diverged from those of Kimuda et al. (40) is that the avidity of IgG specific for SARS-CoV-2, CMV, EBV and RSV did not alter following *M.tb* challenge. However, this may be due to the small sample size given that avidity of PPD-specific IgG also did not show any statistically significant change, and for several antigens there was a trend towards an increase. Furthermore, we used a chaotrope-based avidity ELISA, which has limited ability to detect antibodies of weak or intermediate binding strength, and leads to preferential disruption of conformational epitopes (65). Future studies should evaluate these antibodies using a more sensitive and less-biased method such as surface plasmon resonance (65). If the lack of increase in avidity is validated in larger and more sensitive studies, this may suggest a mechanism other than polyclonal activation of memory B cells, because if the source of the antibodies was indeed memory B cells they would be expected to have undergone affinity maturation and therefore produce antibodies of higher avidity.

That we saw enhanced titres of heterologous antibodies against some but not all pathogens following *M.tb* infection is also inconsistent with polyclonal activation of memory B cells; notably this lack of generalizability is in agreement with the findings of Kimuda et al. who found higher antibody responses to a range of unrelated pathogens but not EBV or adenovirus in active TB patients compared with healthy controls (40). We observed enhanced responses more consistently against the viruses than bacteria evaluated; as better stimulators of B cells, viruses may induce a superior memory B cell pool that is then available for non-specific re-stimulation (66). Kimuda et al. hypothesise that differential effects by pathogen may relate to varied rates of exposure, with more recent or potent exposures to particular pathogens generating new antibody responses that then mask any boost that may otherwise be mediated by mycobacterial-driven activation (40). This could plausibly be the case in our study as responses to *S. abortus* LPS (which is highly conserved across Gram-negative bacteria) and *E. coli* (which is ubiquitous in the environment) were not enhanced.

There are some additional limitations to our study. The antigens were derived from human pathogens but in some cases are highly species-specific and effects may have been clearer if antigens from the respective rhesus macaque homologues were used (for example RhCMV, non-human pneumoviruses and RhLCV which belong to the same lymphocryptovirus genus as EBV). Nonetheless, these are closely-related as viral evolution has paralleled mammalian speciation and extensive antibody cross-reactivity would be expected. While we selected antigens from a range of pathogens including both bacteria and viruses, it is possible that antibody responses against other pathogens differ from those tested. It is notable that most animals had pre-existing antibody responses to SARS-CoV-2 S1 at baseline despite the studies being conducted prior to the COVID-19 pandemic. We hypothesise that these represent cross-reactive antibodies conferred by prior seasonal coronaviruses, as has been widely reported in pre-pandemic human cohorts (67, 68). The animals in Study 3 were older in age (10-12 years) compared with those in the other studies (3-6 years), likely resulting in greater antigen experience and wider repertoire, which may account for the higher baseline antibody responses observed to several of the heterologous pathogens in this group.

In conclusion, we have validated the findings of Kimuda et al. demonstrating that *M.tb* infection, but not BCG vaccination, is associated with increased antibody responses to unrelated pathogens. We have extended this to demonstrate its applicability across both IgM and IgG isotypes, and for a longer period post-BCG vaccination. We also show that the candidate TB vaccine, MTBVAC, like BCG, does not enhance heterologous antibody responses. While further work is required to determine the functional capacity of heterologous antibody responses enhanced by *M.tb* infection and elucidate the underlying immune mechanisms, our capacity to do this was limited by sample availability. Future studies should explore the impact of varying infection dose, duration of infection and the role of chronic inflammation in mediating the responses observed. Our findings nonetheless expand the otherwise very limited literature on mycobacteria and non-specific antibody responses, with potential implications for understanding the increased risk of Burkitt’s lymphoma in TB patients and strategies for targeted prevention or treatment.

## Data availability statement

The raw data supporting the conclusions of this article will be made available by the authors, without undue reservation.

## Ethics statement

The animal study was approved by UK Health Security Agency (and predecessor organisation Public Health England), Porton Down Ethical Review Committee. The study was conducted in accordance with the local legislation and institutional requirements.

## Author contributions

MPPA: Data curation, Formal analysis, Investigation, Methodology, Supervision, Writing – original draft, Writing – review & editing. HJ: Data curation, Investigation, Visualization, Writing – original draft, Writing – review & editing. HRA: Data curation, Investigation, Methodology, Visualization, Writing – original draft, Writing – review & editing. CD: Data curation, Investigation, Visualization, Writing – original draft, Writing – review & editing. AW: Investigation, Writing – original draft, Writing – review & editing. CS: Investigation, Writing – original draft, Writing – review & editing. MD: Investigation, Writing – original draft, Writing – review & editing. SL: Visualization, Writing – original draft, Writing – review & editing. DW: Methodology, Resources, Writing – original draft, Writing – review & editing. EP: Resources, Writing – original draft, Writing – review & editing. SK: Methodology, Writing – original draft, Writing – review & editing. SB-R: Methodology, Writing – original draft, Writing – review & editing. NA: Resources, Writing – original draft, Writing – review & editing. CM: Resources, Writing – original draft, Writing – review & editing. SS: Investigation, Resources, Writing – original draft, Writing – review & editing. HM: Funding acquisition, Project administration, Supervision, Writing – original draft, Writing – review & editing. RT: Methodology, Project administration, Resources, Supervision, Validation, Visualization, Writing – original draft, Writing – review & editing, Conceptualization, Formal analysis, Funding acquisition, Investigation.
